# An Update on CFTR Drug Discovery: Opportunities and Challenges

**DOI:** 10.3390/biom12060792

**Published:** 2022-06-06

**Authors:** Pasqualina D’Ursi, Paola Fossa

**Affiliations:** 1 Institute for Biomedical Technologies, National Research Council (ITB-CNR), 20090 Segrate, Italy; pasqualina.dursi@itb.cnr.it; 2 Department of Pharmacy, Section of Medicinal Chemistry, School of Medical and Pharmaceutical Sciences, University of Genova, 16148 Genova, Italy

The *Biomolecules* Special issue on “An Update on CFTR Drug Discovery: Opportunities and Challenges” includes three original research articles and a webinar session focusing on some recent findings concerning CFTR drug discovery.

Cystic Fibrosis (CF) is a severe autosomal recessive disorder caused by mutations in the cystic fibrosis transmembrane conductance regulator gene encoding the CFTR protein, which is a chloride channel expressed in many epithelial cells. Despite a consistent clinical progress in delaying disease progression with symptomatic and targeted therapies, individuals affected by CFTR develop several chronic complications, which significantly restrict their life expectancy but above all their quality of life. Current therapies rely in a large part on symptomatic treatments.

In this Special Issue, the contribution of Mori et al. [1] described the recombinant production and biochemical characterization of human DNase1L2, a mucolytic enzyme that could represent a valuable alternative for the treatment of CF lung disease. DNase1L2 showed efficient mucolytic activity and, importantly, exhibited enhanced resistance to actin inhibition compared with recombinant human DNase1 (rhDNase). Moreover, a PEGylated variant of DNase1L2 that fully preserved the enzyme activity was obtained.

Parallelly, industry and academia in recent years have carried out great efforts in pursuing drug discovery campaigns targeted to discover effective CFTR modulators, able to improve the deficient or defective activity of the mutated CFTR by either restore its tracking (correctors) or gating (potentiators). Only a few of the discovered compounds have progressed to the clinical trial stage and four of them, plus their combinations, are currently on the market. Near these approved modulators, several investigational ones are under Phase I or Phase II clinical trial or being fully developed and characterized in their molecular mechanism of action. As further development of these studies, the scientific community is engaged in the identification of small molecules able to synergize with lumacaftor, increasing its therapeutic activity. In the present Special Issue, Baroni et al. [2] investigated the putative site of action of FCG, an aminoarylthiazole derivative with proven CFTR corrector activity, and its synergistic effect with lumacaftor. They found that FCG was able to enhance F508del-CFTR total expression and its combination with lumacaftor was able to increase not only the total expression but also the maturation of the mutant protein. Biological tests supported by molecular modelling studies suggest the Nucleotide Binding Domain 2 (NBD2) as the target for FCG activity. Franceschelli et al. [3] investigated how a synergic effect of lumacaftor in combination with matrine enables a significant repair of CFTR trafficking and gating at the apical membrane of the epithelial cell. Based on the inflammatory properties possessed by matrine, they also investigated and reported matrine and lumacaftor synergic ability in counteracting the specific inflammation which characterizes the CF pathology.

Furthermore, the Special Issue included a dedicated webinar (https://biomolecules-5.sciforum.net/) (accessed on 1 June 2022) with the contributions of two eminent scientists in the field, Nicoletta Pedemonte, from Istituto Giannina Gaslini, Genova, Italy and Isabelle Callebaut, from Sorbonne Université, Muséum National d’Histoire Naturelle, Paris, France.

Pedemonte presented and fully discussed the most recent findings on CFTR drug discovery, from approved drugs to the most promising ones in Phase I and II, including new biological tests alternative to bronchial epithelial cells, therapying of rare CF mutations, alternative targets for CFTR therapy. Hot topics have been the importance of identifying therapies for non-responders [4] and of developing new drugs targeted for CFTR orphan mutations.

Callebaut focused on the dynamic aspect of the CFTR protein, exhaustively presenting an understanding of the structural complexity of the native and the mutated F508del-CFTR protein. In addition, the most significant homology models for CF drug discovery were discussed in the light of the recent cryo-electron microscopy data. Once more, Callebaut remarked the interest of the scientific community towards those small molecules able to synergize with lumacaftor, thus improving its rescue of the impaired protein. In particular, she reported the activity of some bis-phosphinic acid derivatives and their implications for CFTR modulation [5].

In conclusion: this collection of work provided a broad view of the complexity of research on CFTR. Near the opportunities offered by the recent discoveries in the field, namely new potential drugs, new biological tests, alternative targets, combo therapies; it underlined the need of better targeting CFTR mutations different from F508del and of solving the non-responders problem, thus expanding CFTR therapeutic treatment to a larger part of patients.
